# Progress in Bacteriology

**Published:** 1901-03-09

**Authors:** 


### PROGRESS IN BACTERIOLOGY.

**Bronchitis.**—Ritchie[1] has conducted a series of researches into the infectious nature of bronchitis. It has long been known that the nasal cavities and the air passages below the glottis are sterile under normal conditions. In the sputa of bronchitics many organisms have been found: these may have been derived from the mouth, but if the bronchial secretions are examined by removing the lungs immediately after death this source of error is avoided. Ritchie made such examinations in cases of fatal bronchitis in children. Forty-nine cases in all were examined, in twenty-two of which pneumonia was also present. Films prepared from the mucus showed micro-organisms in all the cases, and in all but six successful cultures were also made. By far the most common organism was the streptococcus, which was found in thirty-four cases; in pure culture on three occasions. The micro-organisms associated with the streptococcus were: B. coli, 17 times; diplococcus pneumoniæ, 15 times; B. influenzæ, 10 times; staphylococci, 8 times; B. diphtheriæ, 5 times; pseudo-diphtheriæ, 4 times; B. pyocyaneus, 3 times; and an encapsuled bacillus twice. Saprophytic bacteria were associated with the streptococcus in 6 cases, and pathogenic in 19.

Some of the cultures showed the presence of B. coli only, and this was rather to be regarded as a post-mortem invasion than as an exciting cause. Forty-seven per cent. of all cases showed the presence of pneumococci, and twice they were found in pure culture. In nine sporadic cases of influenza the bacillus of Kitasato was found, but never in pure culture. Similarly staphylococci were never found alone, and were never plentiful. They probably represented a secondary infection. The diphtheria bacillus and the pseudo-diphtheria bacillus were found in eight and in six cases respectively, and it is certain that these may excite a bronchial catarrh quite apart from the formation of membrane. The B. pyocyaneus was regarded as the exciting cause in four cases, and an unnamed encapsuled bacillus in three.

**Tubercle Bacillus.**—Pusey[2] records a case of lupus cured by exposure to the Roentgen rays. The patient, a woman aged thirty-eight, had a large patch on the left cheek, and this was subjected to the rays almost daily from May to October with complete recovery. Other observers have met with equally good results, but in view of the researches of Wolfenden and Forbes-Ross[3] it is difficult to account for them. In testing the effects of the Roentgen rays upon various forms of life they found that, as a rule, there was excessive growth and proliferation, and this was especially well marked in the case of the B. tuberculosis. Cress seeds and yeast exposed to the X rays germinated more quickly and more strongly than if not so exposed, and pigment-forming organisms, pyogenic cocci, and B. coli all flourished. The tubercle bacilli grown in the presence of the rays appeared as fat, glossy, evenly staining rods of abnormal size. The effects of chemical, thermic, and electrical agencies could be excluded. Ravenal[4] gives details of three undoubted cases of tuberculosis of the skin, due to inoculation with the bovine tubercle bacillus. This record is of great value in view of the fact that many deny the identity of the two varieties of organisms, and seek to minimise the risk of the transmission of tubercle from lower animals to man.

The fact that tubercle bacilli are immobilised and clumped by the blood of tuberculous persons has led some observers to attempt methods of serum diagnosis similar to Widal's test. The difficulty has been to obtain a culture of bacilli free from clumps and showing any degree of motility. Edsall[5] states that such a culture may be obtained by growing an old attenuated stock culture in glycerin peptone bouillon with daily agitation. In such a medium considerable motility is developed, and the bacilli are freely separate from one another. The culture is incubated from eight to twelve days and the upper portion only used. The dilution of serum he uses is 1 in 20, and this is mixed with the culture in sedimentation tubes. In positive cases the tubes are clarified in from two to twenty-four hours, and if a microscopic examination be made there will be found loss of motility and clamping. The intensity and frequency of agglutination are in inverse relation to the virulence of infection and in direct relation to the resistance of the animal infected. Reviewing the results of Arloing, Courmont, Mongour, and Buard he comes to the conclusion that, although the technique is a difficult one, the test is extremely delicate and will be of the greatest value in obscure cases of tuberculosis.

**Tetanus.**—The outbreak of tetanus in Northern Italy, due to the use of antidiphtheritic serum, has recently been the subject of an official inquiry, and the Italian Sanitary Inspector-General finds that the accident was due to lack of proper precaution in preparing the serum or to insufficient disinfection of the stoppers of the receptacles. A group of four cases of tetanus has also been traced to the use of injections of hæmostatic gelatine in cases of enteric fever at the Santo Spirito Hospital, Rome. As tetanus can only be communicated by the direct inoculation of the bacillus it follows that gross carelessness in the processes of manufacturing must have existed. Thalman[6] has recently investigated the methods by which tetanus may be contracted. His conclusions are as follows:—The bacillus cannot find entry through the healthy skin, or by the mucous membrane of the digestive or urinary tract. In conditions of catarrh the nasal and respiratory passages are ready channels of infection, and these are often probably the seat of inoculation in so-called idiopathic cases. Abrasions of skin or mucous membrane are easily infected. He recommends that the serum treatment be combined with continuous oxygen inhalations and the administration of expectorants.

**B. Coli.**—Owing to the association of this organism with many pathogenic processes much attention is paid to its study at the present time. Dormer Harris[7] thinks that although there may be grades of virulence and toxicity it is more probable that there are a great variety of coliform organisms, some virulent and others non-virulent, and that the only true test of causal relationship is finding the organism in the blood. The diseases excited by the bacillus coli are peritonitis, enteritis, cholera nostras, epidemic diarrhœa, pleurisy and meningitis, and suppuration. A detailed account of the varieties of B. coli with their cultural reactions is given by Ford.[8] This author is of opinion that intestinal organisms have the power of migrating through healthy gut wall, and that failure to demonstrate this fact has been due to imperfect cultural methods. In a paper by McCrae[9] on agglutination tests tried with B. coli communis, it is, too, shown that the coli group embraces many varieties, each one of which may differ in its agglutinating power.

This matter is one of practical importance, for in the bacteriological examination of water the nature of the organisms present, rather than the number, has come to be regarded as the chief point, and the simple presence of B. coli has been held by some to be sufficient to condemn the supply. Linsley and Stone,[10] in examining a series of 509 waters, met with the following varieties of coliform organisms; fifty-six which gave typical reactions; three which fermented sugar but did not form indol; one which fermented sugar and formed indol but did not coagulate milk; three which fermented sugar but did not form indol or coagulate milk; one which formed indol only, having no effect on milk or sugars. Some of these varieties closely approximate to the typhoid type, and would seem to warrant the assertion that the B. typhosus is in reality a pathogenic variety of the B. coli, and that the one may change into the other. However this may be, the fact is evident that the presence of B. coli in water is a very delicate test of sewage pollution—ten times more delicate, in the opinion of the writers, than any chemical test. A good rule as to the condemnation of a water is as follows:—If B. coli is found in 10 cc. of the water, it is contaminated; if in quantities between 10 and 50 c.c., it is doubtful; but if only in quantities above 50 c.c. it may be passed. In this connection it is noteworthy that in the practical treatment of sewage[11] the coke filter beds do not keep back organisms, but that B. coli are plentiful in the effluent as in the untreated sewage.

**Bienstock**[12] finds that the B. coli and B. lactis ærogenes, normal inhabitants of the intestine, play an important part in preventing the decomposition of albuminous food in the gastro-intestinal tract. The chief organism in effecting this decomposition of fibrin is the B. putrificus, and this has its power inhibited in the presence of the two aforementioned varieties. For this reason it may inadvisable to feed infants suffering from intestinal complaints upon sterilised milk, from which, presumably, the B. coli and B. lactis have been eliminated; for by so doing we may increase the fermentation in the gut. Probably each fermentative organism excites its own particular type of digestive disorder, and has its special antagonistic germ; and this may account for the fact that infants passing strongly alkaline diarrhœic stools do best on a carbohydrate diet, while children with acid stools improve on a proteid diet.

[1] Jour. of Path. and Bact., Dec. 1900. [2] Med. News, Nov. 24, 1900. [3] Med Chron., Oct. 1900. [4] Phil. Med. Jour., July 21, 1900. [5] Med. Chron., Oct. 1900. [6] Zeit. f. Hyg., xxxiii. 3, 1900. [7] Jour. of Path. and Bact., Dec. 1900. [8] Mont. Med. Jour., May, 1900. [9] Ibid. [10] Med. Rec., Sept. 1, 1900. [11] Rep. to Lond. County Council. [12] Ann. de l'Inst. Past, Nov. 25, 1900.

